# Gut Dysbiosis and Dietary Interventions in Rheumatoid Arthritis—A Narrative Review

**DOI:** 10.3390/nu16183215

**Published:** 2024-09-23

**Authors:** Estera Bakinowska, Wiktoria Stańska, Kajetan Kiełbowski, Agata Szwedkowicz, Dominika Boboryko, Andrzej Pawlik

**Affiliations:** 1 Department of Physiology, Pomeranian Medical University, 70-111 Szczecin, Poland; esterabakinowska@gmail.com (E.B.); kajetan.kielbowski@onet.pl (K.K.); szwedkowiczagata@gmail.com (A.S.); dominikaboboryko@gmail.com (D.B.); 2 Department of Medical Biology, Medical University of Warsaw, 00-575 Warsaw, Poland; wstanska@gmail.com

**Keywords:** rheumatoid arthritis, gut dysbiosis, inflammation, diet

## Abstract

Rheumatoid arthritis (RA) is a chronic and progressive autoimmune disease. The pathogenesis of RA is complex and involves interactions between articular cells, such as fibroblast-like synoviocytes, and immune cells. These cells secrete pro-inflammatory cytokines, chemokines, metalloproteinases and other molecules that together participate in joint degradation. The current evidence suggests the important immunoregulatory role of the gut microbiome, which can affect susceptibility to diseases and infections. An altered microbiome, a phenomenon known as gut dysbiosis, is associated with the development of inflammatory diseases. Importantly, the profile of the gut microbiome depends on dietary habits. Therefore, dietary elements and interventions can indirectly impact the progression of diseases. This review summarises the evidence on the involvement of gut dysbiosis and diet in the pathogenesis of RA.

## 1. Introduction

Rheumatoid arthritis (RA) is a progressive autoimmune disease that leads to severe functional impairment and a significantly reduced quality of life [1]. Recent estimations suggest that the global prevalence of RA is approximately 0.5% [2]. This condition typically manifests as painful and swelling small joints of the hands and feet. However, the disease also has extra-articular manifestations, which frequently involve subcutaneous nodules and interstitial lung disease [1]. As an autoimmune disorder, autoantibodies can be detected in patients with RA. These antibodies can target rheumatoid factor as well as post-translationally modified proteins (anti-modified protein antibodies [AMPA]). The most widely known antibodies that belong to the AMPA family recognise anticitrullinated protein (ACPA). However, some antibodies also target carbamylated and acetylated proteins [3].

The pathogenesis of RA is complex and involves interactions between articular and immune cells. Fibroblast-like synoviocytes (FLSs) are considered to be the major drivers of the development of RA. Under inflammatory conditions, FLSs have an invasive character: they actively promote inflammation and degrade articular tissue. Importantly, FLSs largely contribute to the formation of a hyperplastic synovium (pannus) [4]. Other cells that have been implicated in the pathogenesis of RA include macrophages, which secrete tumour necrosis factor α (TNFα) and T cells, which are categorised into several subtypes, among which Th17 cells are highly implicated in the progression of the disease [5,6,7].

Researchers have demonstrated that multiple processes in a human body affect inflammation. One of these processes is gut dysbiosis. It is estimated that 100–1000 bacterial species exist in the human intestine [8]. The gut microbiome is involved in the formation of mucus, epithelial regeneration, and intestinal integrity, among other processes. Due to its secretory properties and ability to regulate the immune, neuroendocrine, and circulatory systems, the gut microbiome affects other tissues and systems. Moreover, the diet affects the composition of the gut microbiome [9]; thus, diet could indirectly affect immune responses. This review discusses the role of gut dysbiosis in the pathogenesis of RA and the influence of diet on the progression of RA.

## 2. The Involvement of Gut Microbiome in Regulating the Immune System

Schluter et al. [10] demonstrated that alterations in the gut microbiome are associated with modifications in the immune system. They evaluated the impact of faecal microbiota transplantation (FMT) in patients undergoing hematopoietic cell transplantation. The FMT procedure administered within 100 days of neutrophil engraftment resulted in increased peripheral white blood cell (WBC) counts. More precise analyses revealed that *Faecalibacterium* and *Ruminococcus* are genera associated with a higher number of neutrophils. By contrast, *Clostridium sensu stricto* and *Rothia* are associated with lower neutrophil counts. In another study, the authors found that preventing maturation of the gut microbiome in mice induces changes in the immune system that lead to elevated susceptibility to *Salmonella* infection. These mice show fewer regulatory T cells (Tregs) in the intestine, alongside lower and higher IgA and IgE serum levels, respectively [11]. Furthermore, intestinal microbiota composition affects immune responses that are involved in the progression of inflammatory and autoimmune disorders. For instance, microbiota was found to regulate inflammation in adipose tissue, which was linked with background mechanisms associated with obesity [12]. In addition, the presence of specific bacteria in the intestine has been correlated with inflammatory status in autoimmune diseases [13]. Due to molecular mimicry and the stimulation of autoantibody production, bacteria can enhance the progression of autoimmune reactions [14]. Interestingly, the composition of the gut microbiome also affects the response to immunotherapy in oncological studies [15,16,17], further highlighting an important relationship between the intestinal microbiome and immune responses. These pharmacomicrobiomic mechanisms are also suggested to play an important role in autoimmune diseases, as gut microbiota composition could predict the response to biological treatment [18]. 

## 3. Gut Dysbiosis and Rheumatoid Arthritis

### 3.1. The Contribution of Gut Dysbiosis to Chronic Inflammation

There have been a number of studies regarding the role of gut dysbiosis in the pathogeneses of inflammatory diseases. Recently, the authors of a large meta-analysis examined the association between gut dysbiosis and 14 rheumatic diseases; they considered 92 observational studies. The authors reported that an RA diagnosis is significantly associated with reduced richness of the gut microbiome. There are several differences in bacterial genera in patients with RA, including increased relative abundance of *Streptococcus*, *Veillonella*, *Actnomyces*, and *Eggerthella*, and decreased relative abundance of *Parasutterella*, *Faecalibacterium*, *Megamonas*, *Gemmiger*, *Odoribacter*, and *Fusicatenibacter* [19]. In addition, the richness of the gut microbiome seems to be lower among high-risk patients with RA [20]. Interestingly, the gut microbiome profile seems to change as the disease progresses. For example, *Collinsella aerofaciens* is more abundant in patients with stage 1 RA, *Veillonella parvula* is associated with stage 3 RA, and *Bifidobacterium longum* and *Eggerthella lenta* are associated with stage 4 RA. Interestingly, gut dysbiosis in patients with RA could explain its associations with different diseases. For example, *Bifidobacterium dentium*, a bacterium correlated with the occurrence of periodontitis, is elevated in patients with RA [21]. Taken together, there is ample evidence that the gut microbiome is altered in patients with RA. 

Intestinal permeability is another pathophysiological mechanism that could be associated with RA. The barrier that divides the lumen of the intestine from the systemic circulation is a complex structure comprising a mucus layer, epithelial cells, a vascular endothelium, immune molecules, and substances related to digestion. Altered barrier function and increased permeability have been correlated with the development of various diseases, of which the inflammatory bowel diseases have probably been the most widely investigated [22]. In RA, researchers have demonstrated elevated markers of intestinal permeability. According to a recent analysis, patients with RA present higher levels of faecal zonulin (a marker of permeability); some patients also demonstrate elevated serum levels of zonulin. Importantly, the authors reported a positive correlation between faecal and serum zonulin levels [23]. However, a recent systematic review was inconclusive regarding intestinal permeability in RA cohorts [24]. Perhaps increased permeability affects a proportion of patients with RA under certain conditions or depending on other factors, such as the disease stage, sex, or comorbidities. Interestingly, Luo et al. [20] demonstrated that performing FMT from high-risk patients with RA on mice is associated with enhanced intestinal permeability. It has been reported that a 20-year-old patient with refractory RA was successfully treated with FMT from an 8-year-old child donor. This finding further suggests that FMT may be consider as a therapeutic option for RA patients [25]. However, in mice with severe joint swelling induced by laminarin and oral inoculation of *Porhyromonas gingivalis*, followed by FMT administration, the procedure resulted in a higher arthritis score in mice treated with FMT compared to mice which did not receive FMT [26]. Interestingly, the clinical trial for patients with psoriatic arthritis tested FMT as a therapeutic option. The method was recognised as safe and tolerable for patients. Several adverse events occurred such as nausea and vomiting [27]. However, the other clinical study by Kragsnaes et al. reported that the curative effect was better in the placebo group than in the FMT group, 81% and 40%, respectively [28]. Systemic lupus erythematosus (SLE) is another disease where FMT has been used. In the study by Wang and collaborators, the authors demonstrated that MRL/lpr mice treated with prednisone with FMT had increased genera of *Lactobacillus* and decreased *Alistipes and Ruminococcus*. FMT-treated mice presented lower serum levels of antinuclear antibodies (ANA) and anti-double stranded DNA (dsDNA) antibodies in 16 weeks. FMT may alleviate inflammation of kidney cells and skin lesions. However, the efficacy of the treatment was higher in model mice than the FMT group [29]. Intriguingly, lupus progression was suppressed by short-term FMT treatment in 9–13-week-old MRL/lpr [30].

Furthermore, Audo et al. [31] evaluated intestinal permeability in patients with RA by measuring the blood levels of sCD14 and LPS-binding protein (LBP), which are bacterial products. They found higher levels of these markers in patients with arthritis; moreover, these markers correlated positively with disease activity. Intriguingly, the monitoring of permeability biomarkers could be implemented to detect treatment responders. The authors showed that patients who respond to TNF inhibitors have significantly decreased LBP levels [31]. Cheng et al. [21] also provided evidence that patients with RA show increase intestinal permeability. They characterised the synovial fluid microbiome in patients with stage 4 RA and detected *E. lenta*, *B. longum*, *Prevotella copri*, *Clostridium sporogenes*, *Enterococcus gallinarum*, and *Citrobacter freundii*, among others. Importantly, they also frequently detected these bacteria in faecal samples. 

**Figure 1:**
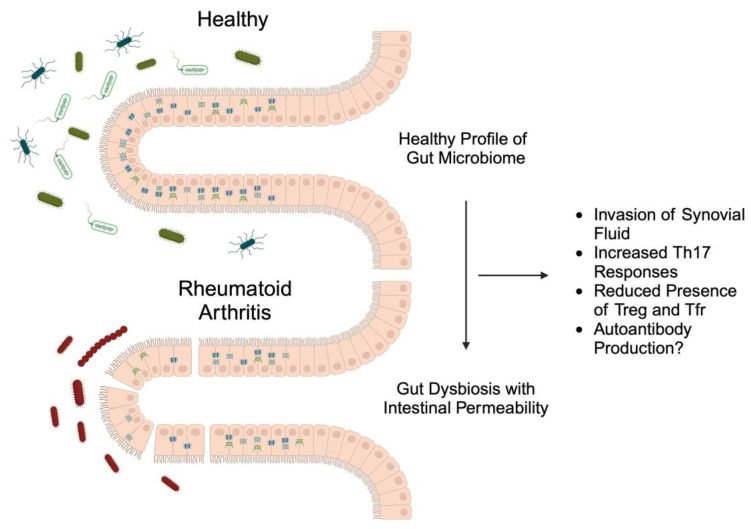
A schematic illustration demonstrating a rich profile of microbiome in healthy people and reduced abundancy of bacteria, together with intestinal permeability in patients with rheumatoid arthritis, which leads to synovial fluid invasion and impaired immune responses. Created with BioRender.com.

As mentioned above, gut dysbiosis affects the immune system. These alterations involve mechanisms associated with RA pathophysiology. RA is strongly associated with the presence and action of Th17 cells. Studies in other disease models have confirmed the ability of gut dysbiosis to increase the number of Th17 cells [32,33]. Gut dysbiosis could also be associated with the Th17 responses observed in RA. *Firmicutes*, one of the phyla whose abundance is reduced in patients with RA, correlates negatively with the presence of Th17 cells [34]. Apart from the altered Th17/Treg ratio, the dysregulation of follicular regulatory T cells (Tfrs) and follicular helper T cells (Tfhs) has been observed. A reduction in Tfrs has been associated with enhanced disease activity and increased production of antibodies. The abundance of Tfrs also depends on the composition of the gut microbiome—for example, it correlates negatively with *Ruminococcus 2* [35]. Regulation of the autoimmune responses could represent another mechanism linking gut dysbiosis with RA disease activity. In other autoimmune disease models, researchers have found that gut dysbiosis is associated with the abundance of autoantibodies [36,37]. Taken together, patients with RA have an altered gut microbiome and potentially impaired intestinal permeability. Consequently, different gut microbiome profiles can alter immune responses, which could contribute to chronic inflammation (Figure 1). 

### 3.2. Bacteria Species Associated with Rheumatoid Arthritis

#### 3.2.1. *Prevotella copri*

*Prevotella copri* represents a species that is extensively explored in the context of RA. Scher et al. [38] revealed that an increase in intestinal *P. copri* correlates positively with the presence of RA. Furthermore, it is associated with reduced diversity of other bacterial species. *P. copri* is found more often in patients with new-onset RA than in those with long-lasting, managed disease. Strains isolated from RA patients demonstrated some differences compared with the reference strain. Thus, identification of *P. copri* strains could have diagnostic potential. These differences imply that particular genes might correspond with the disease. Faecal DNA analysis from patients with new-onset RA and healthy subjects enabled researchers to create a catalogue of 3291 *P. copri* open reading frames (ORFs). Seventeen ORFs were identified only in patients with new-onset RA and two ORFs only in healthy controls. As a result of these findings, differentiation between healthy individuals and those susceptible to RA may be carried out based on the presence of these specific ORFs [38]. Human leucocyte antigen (HLA) class II–associated autoimmune disease is characterised by phenomena in which contact with foreign antigens—gut, oral, or inhaled microbes—is speculated to result in autoimmune reaction. In the case of RA, this leads to joint damage and inflammation [39].

*P. copri* could play a role in the aberrant immune responses involved in the progression of RA. For example, the bacteria could stimulate a pro-inflammatory environment through Th17 cells. Additionally, it causes joint damage via molecular mimicry: the similarity between *P. copri* and joint tissue molecules can lead the immune system to incorrectly destroy healthy cells. The T and B cells from patients with RA target *N*-acetylglucosamine-6-sulfatase (GNS) and filamin A (FLNA) in 52% and 56% of cases, respectively. GNS and FLNA are similar to proteins present in the bacteria *P. Copri*. Particularly, epitopes presented by HLA-DR, possess sequences that match closely those in *P. copri*. This similarity may lead to B cells and T cells falsely attacking autoantigen. Patients with RA have antibodies against both autoantigens and *P. copri*, which implies that initiation of immune response against this bacterium may trigger a response against autoantigens. These antibodies are specific for patients with RA—they are not typically found in healthy subjects or in patients with different diseases [39].

A point mutation in the zeta-chain-associated protein kinase 70 (*ZAP70*) gene predisposes mice to develop RA-like autoimmune arthritis by impairing the function of the T-cell receptor (TCR). ZAP70 is a vital element of the TCR signalling pathway, which in turn is crucial for accurate T cell activation and function. When their activation and function are impaired, T cells become autoreactive. When SKG mice with a point mutation in *ZAP70* are kept in specific pathogen-free (SPF) conditions, which include particular environmental microbes—especially those connected to the gut microbiome, such as *P. copri*—they develop arthritis upon exposure to environmental factors such as fungi. However, when kept in germ-free (GF) conditions, even upon exposure to environmental factors like fungi, they do not develop arthritis. This suggests that the gut microbiome is essential for the activation of autoreactive T cells. Importantly, monocolonisation of *P. copri* augments the Th17 cell responses and arthritis in SKG mice, which is an animal model used to study RA [40]. Moreover, these mice harbour elevated interleukin (IL)-17 responses to the arthritis-related autoantigen 60S ribosomal protein L23a (RPL23A) in lymphocytes located in regional lymph nodes and the colon, but not the spleen. A diet rich in carbohydrates, simple sugars, and fibre is linked to increased abundance of *P. copri* [41].

#### 3.2.2. *Collinsella*

Jeong et al. [42] identified the *Collinsella* genus as the most crucial among the top 20 genera for classifying patients with RA by random forest prediction analysis. *Collinsella* displays notable discrepancy between healthy control subjects and patients with RA. Multiple studies have demonstrated the abundance of *Collinsella* in the gut microbiome of patients with RA [42,43]. *Collinsella* diminishes the expression of tight junction proteins in a human intestinal epithelial cell line, a change that impacts gut permeability [43]. Systemic inflammation might be a consequence of augmented permeability of the intestine because translocated substances may be recognised as foreign. In addition to increased IL-17 due to the activity of *Collinsella*, the chemokines CXCL1, CXCL5, and RORα are also overexpressed. Moreover, nuclear factor kappa B1 (NF-κB1) expression implies activation of inflammatory pathways. Alpha-aminoadipic, asparagine, and IL-17A are associated with high prevalence of *Collinsella* [43]. These metabolites are most likely produced by *Collinsella* spp. and influence gut permeability. Alpha-aminoadipic acid is an indicator of autoimmunity and changes in human collagen with ageing. Asparagine is an amino-acid that plays a role in blocking apoptosis and the tricarboxylic acid cycle. Changes to collagen and blocking apoptosis may be involved in the pathogenesis of RA, but this potentiality requires investigation [43]. Moreover, *Collinsella* is linked to arginine-deiminase activity, which indicates its role in protein citrullination and development of RA [44].

#### 3.2.3. *Lactobacillus*

*Lactobacillus casei*, *Lactobacillus rhamnosus*, and *Lactococcus lactis* are suggested to play a protective role in RA [45,46,47,48]. *L. casei* supplementation tends to lower *C*-reactive protein (CRP) levels and the number of tender and swollen joints and induces anti-inflammatory responses. Specifically, *L. casei* enhances IL-10 levels and reduce IL-6 and TNF-α levels. Supplementation with *L. rhamnosus* increases IL-1 but has no impact on IL-6, IL-10, IL-12, and TNF-α. *Ligilactobacillus salivarius* most likely induces both pro- and anti-inflammatory responses, depending on the context. It is more frequently found elevated in patients with RA and is abundant in very aggressive cases of RA [49,50,51]. However, Liu et al. [49] revealed that *L. salivarius* alleviates the inflammatory symptoms and reduces bone erosion and neutrophil infiltration in a mouse model of collagen-induced arthritis. The authors speculated that *L. salivarius* prevents disease in healthy individuals but negatively influences immune homeostasis in susceptible subjects. An increased abundance of lactobacilli in patients with RA may lead to progression of the disease. In addition, an imbalance between anti- and pro-inflammatory cytokines may induce autoimmunity.

#### 3.2.4. Depletion of Beneficial Bacteria 

Patients with RA have a markedly altered gut microbiome compared with healthy controls, including a decreased abundance of the genera *Faecalibacterium* and *Streptococcus* and the species *Roseburia inulinivorans*, *Faecalibacterium prausnitzii*, *Bacteroides* spp. [52], and *Parabacteroides distasonis* [53]. However, at the beginning of RA, *P. distasonis* seems to be relatively enriched. *P. distasonis* supplementation improves that state of patients with RA. *F. prausnitzii* decreases joint tissue damage, the arthritis score, and the number of systemic immune cells that release IL-17. Moreover, it influences the composition of the gut microbiome and induces changes in short-chain fatty acid (SCFA) concentrations. The levels of IL-17, IL-1β and TNF-α are decreased in mice treated with *F. prausnitzii*. In addition, those mice show increased abundance of the genera *Bilophila*, *Akkermansia*, *Roseburia*, *Coprococcus*, *Oscillospira*, *Ruminococcus*, and *Clostridium*. *Akkermansia* and *Bilophila* are associated with diminished pro-inflammatory cytokine production. Conversely, *Desulfovibrio* and *Bacteroides* abundance are reduced; these genera are linked with augmented pro-inflammatory cytokine production. Hence, *F. prausnitzii* may have a potential therapeutic role in RA [54].

*P. distasonis* abundance is typically low in patients with new-onset or long-term RA. Oral treatment with live *P. distasonis* has a notable positive impact on RA symptoms in mice. Lithocholic acid (LCA), isolithocholic acid (isoLCA), deoxycholic acid (DCA), and 3-oxolithocholic acid (3-oxoLCA)—metabolites of live *P. distasonis*—alleviate RA symptoms. In particular, 3-oxoLCA and isoLCA act as Takeda G protein-coupled receptor 5 (TGR5) agonists to promote the anti-inflammatory M2 macrophage polarisation and inhibit Th17 cell differentiation. Moreover, 3-oxoLCA and isoLCA suppressed the expression of retinoic acid receptor (RAR)-related orphan receptor-γt (RORγt), which is a marker of Th17 cells [53].

## 4. Dietary Factors Involved in RA Pathogenesis

### 4.1. Fibre

Diet is an essential dietary factor, but it also acts as environmental trigger. Researchers have reported a dramatic increase in the frequency of autoimmune diseases in developed countries, and some of them suggest that the Western diet could contribute to this phenomenon [55].

Fibres are complex carbohydrates consisting of soluble and insoluble components. Due to the lack of digestive enzymes, humans are not capable of digesting fibre, and this inability is associated with its various beneficial effects [56]. The recommended daily allowance for fibre intake is 38 g/day for men and 25 g/day for women [56]. Insoluble fibre passes through the digestive tract intact. In the small intestine, soluble fibre is also resistant to hydrolysis, but it is fermented in the large intestine by gut microbiota into SCFAs. The three main SCFAs present in the human body are acetate, propionate, and butyrate. SCFAs are the most abundant of the physiologically active metabolites of fibre: they are sources of energy for the gut microbiota and contribute to changes in the composition of the gut microbiome [55,56]. They enhance intestinal barrier integrity, epithelial cell turnover and the production of antimicrobial elements. For instance, butyrate stimulates the expression of tight junctions, which promotes barrier integrity. Furthermore, SCFAs have immunomodulatory properties, as they can suppress IL-12 production in dendritic cells (DCs) and enhance Treg presence [57]. The relation of SCFAs with bacteria is bidirectional, as the gut microbiota also impacts the level of production of SCFAs. Specifically, members of *Clostridium*, *Eubacterium*, *Butyrivibrio* genera are, among others, responsible for producing SCFAs in the intestine. Butyrate is produced by Clostridial clusters IV and XIVa with high efficiency. Species that dominate in these clusters are *Faecalibacterium pranusnitzii*, *Roseburia* spp. *Anaerostipes*, and *Eubacterium* [57].

A study examining the fibre intake among patients with RA revealed low consumption of dietary fibre: 98% of study participants consumed less than 30 g fibre/day [58]. The authors of multiple studies have found that a suboptimal fibre intake increases the prevalence of RA by up to 25% [59,60]. A notable inverse correlation between total fibre intake and RA emergence has been found, pointing to cereals as a valuable source of fibre [61]. Hager et al. [55] reported that patients who consumed daily high-fibre bars or cereals for 28 days showed a significant improvement in their physical and mental quality of life, as measured by the Short Form 36 Health Survey. 

As mentioned previously, the Th17/Treg ratio is dysregulated in RA. Importantly, fibre intake is associated with an increase in the number of Tregs. Mechanistically, fibre enhances T cell polarisation rather than the total number of T cells [55]. Another immunological effect of fibre occurs via a decrease in IgA antibodies. Interestingly, Hager and colleagues showed that the decrease was related to the global IgA presence and not specific to the ACPA [55]. Nevertheless, serum IgA ACPA is associated with the activity of RA [62]. Therefore, the impact of fibre intake on the total abundancy of IgA antibodies could suggest its positive effects on immunity. 

The intestinal barrier is important in maintaining homeostasis, and it seems to play an essential role in the development of autoimmune conditions. Zonulin, which controls intestinal permeability, and calprotectin, a marker of inflammation in the gut, are reduced in the serum of individuals who consume a high-fibre diet in the short term [55]. Furthermore, low levels of fibre may decrease the intestinal mucus level, which suggests that fibre dictates the profile of the intestinal bacteria that metabolise mucus [63]. Mucus degradation releases free sulphates that can then be used by sulphate-reducing bacteria. As a result, microbes may reach the epithelium and activate enterocytes by interacting with Toll-like receptors (TLRs), subsequently activating NF-κB, type I interferon (IFN), and other inflammatory pathways, which leads to the production of TNF-α, IL-1β, IL-6, IFN, and IL-17, and activation of the immune system [63].

Among patients with RA, 50% of mortality is attributed to cardiovascular events. As fibre intake reduces the risk of obesity, diabetes, hypertension, and other cardiovascular diseases (CVDs), high-fibre diets can be especially beneficial in patients with RA [58]. Consistently, there is evidence that an increased risk of cardiovascular mortality correlates negatively with dietary fibre intake due to the hypocholesterolaemic effects of fibre [56]. The positive effect of fibre on the course of RA is mainly mediated by its metabolites, SFCAs. Indeed, oral administration of butyrate ameliorates experimental RA in mice due to increased IL-10 production by B cells [64,65]. Similarly, propionate administration in the drinking water increases the number of Tregs and IL-10 production [66].

Intriguingly, the effect of fibre also depends on the gut microbiome. Colonisation of the murine intestine with *P. copri* and feeding mice with a fibre-containing diet leads to a more severe inflammatory response. In addition, these mice display a more severe course of the disease denoted by a higher arthritis score and enhanced joint damage. This fibre-rich diet also increases the expression of matrix metalloproteinase-3 (MMP-3) and cathepsin 1 (Cts1), which play a role in joint destruction in RA. The detrimental effect of *P. copri* in the context of high fibre intake may be explained by the ability of this bacterium to activate the TLR4 pathway, thus augmenting the immune response. As mentioned previously, *P. copri* exhibits marked genomic diversity: different isolates have unique features. The strain isolated from the patients with RA in this study expresses rhamnogalacturonan-II degradation-related enzymes, which allows it to metabolise glycan present in the human diet. Consequently, it produces pro-inflammatory metabolites from fibre, such as fumarate and succinate [67].

### 4.2. Omega-3 Fatty Acids

*N*-3 polyunsaturated fatty acids (PUFAs) are a part of the fatty acid family, that are characterised by containing a final double bond between carbons 3 and 4. The main components are eicosapentaenoic (EPA), docosapentaenoic (DPA), alpha-linoleic acid (ALA), and docosahexaenoic acid (DHA) acid. PUFAs are found in the flesh of both lean and oily fish, as well as in supplements, such as fish oil [68]. These molecules are well-recognised anti-inflammatory and immunomodulatory agents, decreasing TNF-α, IL-1 β, IL-6, and cyclooxygenase-2 (COX-2) metabolites [68,69]. Moreover, they have vasodilatory, bronchodilatory and anti-aggregatory properties [68]. 

Humans evolved on a diet with the *n*-6/*n*-3 PUFAs ratio about 1. In the Western Diet, the ratio skyrocketed to 15.9/1 [68]. Therefore, with a rising incidence of autoimmune diseases in developed countries, it is speculated that changing dietary habits can be the reason [68]. The inflammatory response exerted by *n*-6 PUFAs is still poorly understood. Thus, due to the current knowledge in this matter, a more significant role is the *n*-6 to *n*-3 PUFAs ratio, which still needs more evaluation [70]. Several studies have proved the positive effect of *n*-3 PUFAs on the course of RA. Specifically, researchers have reported a decrease in the Disease Activity Score 28 (DAS28) score, morning stiffness, the number of tender joints, and the visual analogue scale score (VAS), as well as improvement in the severity of pain [71,72,73]. Moreover, the inflammatory parameters also seem to be affected by the supplementation of PUFAs, with a marked drop in the erythrocyte sedimentation rate (ESR) and IL-1β levels [71,73]. Galarraga et al. [74] observed that 59% of the patients treated with *n*-3 PUFAs could reduce their daily nonsteroidal anti-inflammatory drug requirement by an average of 40%. Animal and human studies have shown that oral treatment with *n*-3 PUFAs is associated with a lower incidence and severity of arthritis [75,76]. A diet high in omega-3 fatty acids is associated with a 15–35% lower risk of developing RA [77,78,79]. However, caution is advised regarding the dosage of omega-3 fatty acids, as these molecules can enhance bleeding. 

PUFAs exert anti-inflammatory effects in several ways. First, they interfere with the production of pro-inflammatory prostaglandins and leukotrienes from arachidonic acid [71]. *n*-3 PUFAs replace arachidonic acid in the cell membrane, thus altering gene transcription, signalling, and metabolism, resolving inflammation [71]. Specialised pro-resolving lipid mediators (SPMs) play a central role in resolving inflammation, protecting the organism from uncontrolled inflammatory processes, and *n*-3 PUFAs act as substrates for their synthesis. SPMs limit granulocyte chemotaxis and infiltration, leading to M2 macrophage polarisation, promoting the Treg response and the release of IL-10, and reducing the release of TNF-α and IL-1β [80]. Based on in vitro experiments, omega-3 fatty acids decrease the expression of adhesion molecules on the cell surface [69]. The production of inflammatory proteins depends on the pro-inflammatory NF-κB pathway [68]. *n*-3 PUFAs activate PPAR-γ and bind to the G-protein-coupled cell-membrane receptor (GPR120), which inhibits the nuclear translocation of NF-κB [81]. 

There is a scarcity of literature regarding the role of omega-3 fatty acids in the modulation of microbiota in RA patients. Animal studies proved that supplementation with EPA/DHA resulted in an increase in abundance of *Actinobacteria*, with a reduction of *Proteobacteria* and *Akkermansia* [82]. Furthermore, rabbits fed with fish oil had higher levels of intestinal *Bacteroidetes* [83]. There are many discrepancies between studies in regard to the effect of PUFAs on particular genera. Some human studies showed that DHA/EPA supplementation resulted in an increase in *Akkermansiaceae* families, *Bifidobacterium* and *Roseburia*, reducing at the same time *Coprococcus*, and *Facelibacterium* genera [84]. Other studies demonstrated a decrease in *Firmicutes/Bacteroidetes*, with an increase in *Prevotella* genus [85]. Others showed an increase in phylum *Firmicutes*, and *Roseburia*, but decrease in *Bacteroidetes* and *Faecalibacterium* [86]. As the results of the current studies are inconclusive, studying the changes in microbiota upon omega-3 fatty acid supplementation is a promising direction for future research. 

### 4.3. Vitamin D3

Vitamin D is a secosteroid that occurs in two forms: vitamin D2, which can be obtained from the diet, and vitamin D3, which is synthesised in human skin in the presence of ultraviolet (UV) light [87]. Known for its immunomodulatory, antibacterial, and antiviral properties, vitamin D has also received attention due to tis possible implications in alleviating chronic inflammatory diseases [87].

Estimating the daily recommended dose of vitamin D is challenging because the definition of vitamin D sufficiency is based on its effect on bones [88]. The optimal serum level of 25-hydroxyvitamin D for immune function has yet to be established. Nevertheless, researchers have found that patients with RA have significantly lower 25-hydroxyvitamin D serum levels [89,90]. However, they found no significant difference between the severity of the RA course and vitamin D serum levels [89]. Recently, researchers analysed the effects of vitamin D (2000 IV/day) in 25,871 participants. A 5-year supplementation was associated with a 40% lower incidence of RA [91]. In another study, treatment with either calcitriol or 22-oxa-calcitriol could reduce swollen joints, pain, morning stiffness, and CRP levels, and improve the ESR score. Moreover, younger female patients showed greater improvement than older male patients [92]. The authors justified the use of 22-oxa-calcitriol based on its lower hypercalcaemic activity compared to calcitriol. Vitamin D supplementation has been reported to significantly lower the DAS28-CRP score [93]. Moreover, researchers have reported a significant inverse association between 1,25-dihydroxyvitamin D, the active metabolite of vitamin D, and the DAS28-CRP score, CRP levels, and the VAS score [94]. Another study failed to find a correlation between the level of vitamin D and disease activity, but did show a relationship with pain relief [95].

Vitamin D plays a role in many processes. In the intestine, it regulates tight junctions and the apoptosis of intestinal epithelial cells, stabilising the gut barrier [87]. It also downregulates TLR2 and TLR4 in monocytes, modulating the response to pathogen-associated molecular patterns (PAMPs) and damage-associated molecular patterns (DAMPs) [87]. The role of vitamin D is also crucial in regulating the inflammatory response by suppressing Th17 and Th1 cells and promoting differentiation of Th2 cells and Tregs [87].

In addition to immune cells, macrophages, DCs, and T cells are also capable of converting 25-hydroxyvitamin D to 1,25-dihydroxyvitamin D [88]. Low 25-hydroxyvitamin D serum levels are suggested to compromise the synthesis of 1,25-dihydroxyvitamin D within immune cells, altering their immune function, dysregulating innate immunity, and leading to over-exuberant inflammatory adaptive immunity [88]. Interestingly, the vitamin D receptor is expressed in activated, not resting lymphocytes, strongly suggesting the role of vitamin D in the inflammatory processes [88].

Studies have confirmed that both active and inactivated forms of vitamin D suppress DC maturation as well as antigen presentation via CD80 and CD86 on monocyte-derived DCs [88], resulting in impaired T cell activation [88]. T cells can also be suppressed by direct inhibition of IL-2 expression by vitamin D [91]. Moreover, 1,25-dihydroxyvitamin D has a more versatile effect on DCs: under the influence of 1,25-dihydroxyvitamin D these cells exhibit an immature phenotype, which promotes Treg development [88]. T cells activated by 25-hydroxyvitamin D– or 1,25-dihydroxyvitamin D–induced tolerogenic DCs exhibit decreased expression of IFN-γ, IL-17, and IL-21. It indicates the suppression of inflammatory Th1 and Th17 cells as well as follicular B helper T (Thf) cells [88]. The production of IL-6, an important factor that stimulates Th17 cells, by CD4+ T cells is also hindered by vitamin D [91]. 1,25-Dihydroxyvitamin D also inhibits autoantibody production by B cells, and promotes monocyte differentiation into macrophages, leading to reduced antigen presentation capacity by decreasing the expression of major histocompatibility class (MHC) II [91].

To the best of our knowledge, the impact of vitamin D supplementation on gut microbiota in RA patients has not yet been investigated. However, a more general role of vitamin D in modulating the microbiome has been examined. Human-based studies found that vitamin D supplementation was negatively associated with the abundance of *Prevotella*. Moreover, it increased the abundance of *Bacterioides* [96]. However, the results are inconsistent as some studies showed individuals with a higher vitamin D intake had greater abundance of faecal *Prevotella*, and reduced *Veillonella* [97]. In patients with untreated multiple sclerosis (MS), supplementation increased the presence of *Faecalibacterium*, *Akkermansia* and *Coprococcus*, as compared to treated MS patients and healthy controls [98]. Other studies did not find any changes in the stool before and after vitamin D supplementation. This may also imply that faecal analysis may not be an appropriate means of studying the effects of vitamin D on gut microbiota [99,100]. An interesting matter is that bacteria themselves have the ability to hydroxylate vitamin D, which sheds a new light on the role of gut microbiota in maintaining the optimal level of this vitamin [101].

### 4.4. Vitamin E

Vitamin E is a term that encompasses tocopherols and tocotrienols, which are available in sunflower, safflower, corn, walnut, and wheat germ oils [102]. It is a potent antioxidant, claimed to be one of the most effective nutrients modulating immune function. Although vitamin E deficiency is rare, an insufficient amount in the body severely alters the normal function of the immune cells [103]. Exceeding the dietary recommendations of vitamin E is speculated to enhance the function of the immune system [103].

The chronic inflammation associated with RA leads to lower alpha-tocopherol serum levels [104,105,106,107]. Moreover, there are reduced levels of vitamin E in the synovial fluid of patients with RA [108,109,110]. Vitamin E may be consumed within the inflamed joint. Whether there is a correlation between vitamin E in the blood and the risk of RA is a controversial issue. Some cohort studies have found an inverse relation [111,112]; however, a large randomised double-blind placebo-controlled trial showed no significant association between vitamin E supplementation and a reduction in the risk of developing RA [113]. However, the trial included a relatively small number of incident cases of RA, and the participants could have been healthier than the general population: they were female health professionals, who are better educated and have a relatively high socioeconomic status [113]. An extensive meta-analysis showed that oral treatment with vitamin E markedly improves morning stiffness, oedema, pain, the DAS28 score, the ESR, and rheumatoid factor levels [114,115]. Combination of vitamin E with conjugated linoleic acids reduces the DAS28 score, pain, and morning stiffness [116]. In another study, the authors found that vitamin E supplementation is not associated with improved inflammatory or clinical parameters, but it is associated with pain reduction [117].

This beneficial action of vitamin E stems from modulating T cell function via incorporation into the membrane of immune cells, maintaining its integrity and participating in signal transduction, cell division, and the generation of inflammatory mediators [103]. When integrated in the cell membrane, PUFAs are protected from oxidation (i.e., vitamin E can scavenge reactive oxygen species [ROS] to prevent PUFA oxidation) [103]. Animal and human studies have shown that a deficiency of vitamin E exerts a negative impact on humoral and cell-mediated immune functions. Fortunately, the changes can be reversed by vitamin E supplementation [103], improving T cell–mediated functions, especially thymic T cell differentiation, lymphocyte proliferation, Th cell activity, and macrophage phagocytic activity. Vitamin E modulates the deterioration of the immune inflammatory response associated with age [103]. Vitamin E administration might be beneficial in autoimmune diseases, especially because it has a particular antioxidant effect on naïve T cells, not memory T cells, possibly due to their greater susceptibility to oxidative damage. Vitamin E enhances early events of T cell activation and even affects the formatting of the synapses between naïve helper T cells and cells presenting antigens [103], thereby preventing the origin of autoreactive T cells.

Although there are limited data—and thus more research is necessary—vitamin E seems to promote gut barrier integrity, supporting favourable changes in the gut microbiome and mitigating the depletion of butyrate-producing bacteria [115,118]. DCs, which are effective antigen-presenting cells, are involved in tolerance and autoimmunity. Specifically, they promote the generation of Tregs and induce T cell non-responsiveness [102]. Studies have shown that vitamin E has the potential to regulate the functions and maturation of DCs, therefore influencing the course of autoimmune diseases [102].

### 4.5. Selenium

Selenium is an essential trace element present in selenoproteins; it is a component of the active centre of over 25 antioxidant enzymes. The most important selenoprotein is glutathione peroxidase, which could have a modulating effect on inflammatory processes in RA. Consistently, a decrease in the serum levels of trace elements is frequently observed in patients with autoimmune diseases [119]. Two cross-sectional studies on patients with RA in New Zealand and Brazil populations led to the conclusion that patients with RA have a selenium intake that is lower than the daily recommended intake [120,121]. However, Yang et al. [122] drew the opposite conclusion. Multiple studies from the 1990s reported lower concentrations of selenium in the serum of patients with RA, compared with apparently healthy individuals [119]. This has been confirmed in a recent meta-analysis, which revealed a significant difference in serum selenium between patients with RA and healthy controls [123,124]. Moreover, low selenium levels have been detected in synovial fluid, erythrocytes, and leucocytes of patients with RA [124,125]. Selenium plays a role in RA. Indeed, successful treatment with corticosteroids, hydroxychloroquine, methotrexate, and anti-TNF agents markedly increase the selenium blood levels, but only in individuals with low baseline levels of this element [119].

Some studies have shown that low selenium may be a risk factor for rheumatoid factor–negative RA [111]. However, the data are conflicting [126]. There have been relatively few studies evaluating the impact of selenium as an adjuvant therapy in RA. In one study, twice daily administration of selenium (200 µg) improved the general health score (a measure of disease activity), the Health Assessment Questionnaire score (a measure of physical performance), and the VAS score (a measure of pain severity) [127]. However, the effect of selenium on RA severity seems to be dose-dependent because the authors of two other studies reported that selenium supplementation did not result in a clinical improvement [128,129]. However, the authors of one of those studies observed a decrease in CRP levels after selenium supplementation [129].

Low selenium blood levels may result from a redistribution of this element, under the influence of proinflammatory cytokines, to tissues for defence purposes [119]. On the other hand, inflammation induces the turnover of selenoproteins, leading to the depletion of selenium [119]. Moreover, selenium seems to act as a negative acute-phase reactant, as an increase in CRP reduces selenium serum levels [119]. In turn, low selenium levels deplete circulating antioxidants, inevitably exacerbating the inflammatory state of the disease, as reactive oxygen species (ROS) production cannot be mitigated [119].

In the synovium, macrophages and activated T cells produce inflammatory cytokines, mainly IL-1β and TNF-α, which induce ROS production. In addition, increased intra-articular pressure results in hypoxia and ROS generation in the joints [119], which eventually leads to chondrocyte apoptosis and damage to cartilage [127]. The antioxidant properties of selenium come from its ability to inhibit the NF-κB cascade, thus reducing ROS inflammation [119]. 

Selenium also plays an important role in regulating the innate and adaptive immune responses. Selenoproteins can enhance INF-γ production, supporting the differentiation and proliferation of T cells, and maintaining antibody levels [119]. Selenium works as an immunostimulant, activating natural killer cells and cytotoxic lymphocyte-mediated tumour cytotoxicity [124].

Animal studies showed that supplementation with selenium resulted in an increase in microbiota diversity, increasing the relative abundance of *Lactobacillus*, *Bifidobacterium*, and *Desulfivibrio*, and reducing that of *Lachnospiraceae*, *Rikinella*, and *Helicobacter* [130]. Some studies also noted an increase in *Dorea*, *Turicibacter*, *Akkermansia*, and a decrease in the level of *Mucispirillum* [131]. One study on mice with breast cancer who were put on selenium supplementation, concomitantly with a high-fat diet, observed altered composition, as well as diversity of gut microbiota. Namely, an increase in the abundance *of Proteobacteria*, *Actinobacteria*, *Akkermansia muciniphila*, *Verrucomicrobia phyla*, *Helicobacter ganmani*, and *Helicobacter japonicus.* Phyla such as *Bacteroidetes*, *Firmicutes*, *Deferribacteries*, *and Spirochaetes* and species such as *Prevotella*, *Muribacalum intestinale*, and *Lactobacillus murinus*, were reduced [132].

### 4.6. Red Meat

The presumption that red meat has an impact on the course of RA has been around for over 100 years [133]. According to contemporary studies, eating > 100 g/day of red meat is associated with a 2-year earlier onset of RA [134]. In a study from Kuwait, patients with active RA had markedly higher consumption of red meat, along with butter, soft drinks, and pastries [135]. Even the large USA National Health and Nutrition Examination Survey (NHANES) has seemingly confirmed the detrimental effect of red meat intake on RA: the authors reported a relationship between beef intake and RA prevalence. Specifically, there is a significant increase in the prevalence of RA with consumption of only 50 g of beef per day [136]. This finding was confirmed in one study [137], but not in two other studies [138,139]. Moreover, the outcome of biological disease-modifying treatment was found to be dependent on dietary factors [136]. A diet high in fibre and low in meat resulted in 82% responses, while a low-fat and high-meat diet had a response rate of only 35%. The negative effect of red meat on RA is possibly a result of advanced glycation end-products, which are formed during cooking meat at high temperatures. These advanced glycation end-products increase inflammation in several diseases, including RA [140].

Red meat contains oxymyoglobin, deoxymyoglobin, and oxyhaemoglobin. During digestion, haem proteins are hydrolysed to amino acids, peptides, and haem groups. The iron in the haem is coordinated with strong ligands: sulphur, nitrogen, and oxygen of amino acids, peptides, and other biological components. Subsequently, free and coordinated haem catalyses oxidative reactions, damaging lipids, proteins, and nucleic acids. It also causes lipid peroxidation and can initiate an oxidative chain reaction, causing biochemical and cellular damage. The degree of damage caused by haem-catalysed oxidation is comparable to that from ionising radiation [141]. This oxidative damage is present in many diseases, including RA. Meat also contains organic sulphur and, when it is processed, sulphate additives. As mentioned earlier, sulphur is a source of energy for sulphate-reducing bacteria such as *Bilophila wadsworthia*, which produces hydrogen sulphide, contributing to the degradation of mucus, and leaving the mucus layer exposed to bacterial penetration [63].

### 4.7. Saturated Fats

Saturated fatty acids (SFAs) are a heterogeneous group of fatty acids that contain only carbon-to-carbon single bonds. They differ in chain length, and their main dietary sources are dairy fats, red meat, and plant oils [142]. According to the current knowledge, diets rich in SFAs contribute to many diseases, including autoimmune diseases, by influencing inflammatory pathways [143]. A study using Mendelian randomisation analyses showed a positive causal effect of SFAs on the incidence of RA. An increase in SFAs elevates the risk of RA [144]. Researchers have reported elevated total SFA and hexadecenoic acid (C16:0) levels in serum/plasma as well as in the synovial fluid of patients with RA compared with healthy controls [145,146,147]. Moreover, there is a significant correlation between C16:0 and an elevated risk of RA and CRP [148].

Even though the detrimental effects of SFAs have been known for a long time, the mechanism underlying them still requires further investigation. Nevertheless, the American College of Rheumatology recommends a Mediterranean diet with low SFA intake [149]. SFAs promote T cell activation and differentiation towards Th1 and Th17 cells, changes that are not favourable for the course of RA [144]. Moreover, hexadecenoic acid activates the STAT5-PI3K/Akt signalling pathway in T cells, which leads to the upregulation of signal lymphocyte-activating molecule family member 3, and proinflammatory cytokines such as TNF-α, IL-1β, IL-2, and IL-6 [150].

SFAs also impact macrophages, essential players in RA pathology. These molecules affect their adhesion, phagocytic capacity, ROS production, and cytokine release [151]. SFAs are potent inducers of TNF-α expression by mouse macrophages under basal and inflammatory conditions [151]. TNF-α is an important molecule in RA, as mice that overexpress human TNF-α rapidly and spontaneously develop severe arthritis [152]. Excessive SFAs in food can enhance pro-inflammatory conditions by activating TLR4 [144,151]. Haversen et al. [153] proved that the SFA palmitic acid stimulates TNF-α, IL-8, and IL-1β production.

Dietary SFAs result in significant changes in the composition of the gut microbiome, including a decrease in *Bifidobacteria*, *Eubacterium*, rectal *Clostridium coccoides*, and *Bacteroides*. These changes lead to a shift to a dominance by Gram-negative bacteria in the intestine, and an increase in lipopolysaccharide (LPS). TLRs in the cell membranes recognise LPS that is circulating in the bloodstream and activate NF-κB, increasing the expression of inflammatory genes. SFAs themselves also trigger the proinflammatory signalling pathways by interacting with membrane receptors [154] (Table 1). 

## 5. Dietary Interventions for RA Management

One of the best-studied and most frequently recommended diets for RA is the Mediterranean diet. It is a source of numerous polyphenols and vitamins, and its protective effects against CVDs, cancer, and diabetes are well known [155]. The foundation of the Mediterranean diet is olive oil, which is a source of unsaturated fatty acids, fresh fruits and vegetables, legumes, nuts, and fish. It also involves limiting the consumption of highly processed foods and those that are sources of refined carbohydrates or SFAs (e.g., red meat). In the 2022 American College of Rheumatology (ACR) guidelines, the Mediterranean-style diet is conditionally recommended for individuals with RA, unlike other formally defined diets. However, the level of certainty of the evidence is assessed as low to moderate [156]. Based on the French E3N-EPIC study, which includes a large cohort of women followed since 1990, adherence to the Mediterranean diet may reduce the high risk of developing RA in women who smoke. However, this finding requires further research [157]. In a study from Kuwait, the Mediterranean diet, alongside optimal pharmacological therapy, had a supportive effect on individuals with RA, reflected by a DAS28 score that remained below 3.2 in those adhering to the diet [135]. On the other hand, in a cross-sectional study of 184 Iranian patients, there was no association between adherence to a Mediterranean-style diet and RA activity [158]. There were interesting findings in a cross-sectional study involving patients from the NHANES database, which described the potential benefit for improving mental health in individuals with RA. Adherence to the Mediterranean diet is associated with a lower risk of developing depression in patients with RA. The authors emphasised the need for more rigorous cohort studies in the future to clarify the exact causal relationship [159].

In other studies, vegetarian and Mediterranean diets were shown to attenuate disease activity in patients with RA, showing improvement in a number of tender and swollen joints, Ritchie’s articular index, the pain score, the duration of morning stiffness, grip strength, the ESR, CRP levels, and WBC counts [160,161]. Interestingly, both vegetarian and Mediterranean diets are high in fibre, therefore increasing SCFA levels [162]. A clinical trial evaluated patients who followed an uncooked vegan diet called living food (LF). Such a diet is rich in vitamin E; polyphenols such as quercetin, myricetin, and kaempferol; and fibre. The participants experienced decreased joint stiffness, morning stiffness, and pain at rest, and an improvement in self-experienced health. Because the LF diet has a positive impact on the gut microbiome, it is suggested the improvement of RA is due to the microbiologic effect [163].

It has been shown that the Mediterranean diet may also have a beneficial impact on the gut microbiome. It affects the composition, metabolic activity, and diversity of the gut microbiome, as reflected in the results showing higher production of total SCFA [164]. It is considered that the Mediterranean diet promotes the growth of bacteria such as *Bacteroides* spp., *Lactobacillus* spp., and *Bifidobacterium* spp., while simultaneously inhibiting the development of *Firmicutes* and *Proteobacterium* [165]. This relationship is reflected in the context of RA; an Italian study conducted by Picchianti-Diamanti et al. confirmed that strict adherence to the Mediterranean diet in individuals with RA leads to an increase in *Bacteroides fragilis*, which may reduce disease activity by inhibiting inflammation and suppressing autoimmunity through the induction of Treg cells [166].

Other dietary models have also been evaluated. In 2017–2018, an anti-inflammatory diet similar to the Mediterranean diet was evaluated in the randomised, controlled crossover Anti-Inflammatory Diet in Rheumatoid Arthritis (ADRIA) trial in Sweden [167]. The principles of this diet resemble those of Mediterranean-style diets, including limiting meat consumption to ≤3 times per week in favour of eating fish (mainly salmon). Additionally, the participants received the probiotic *Lactobacillus plantarum* 299v with breakfast 5 days a week. In the primary analyses, there was a clinically significant decrease in the DAS28 score; however, there was a reduction in disease activity in the uncorrected analyses. Other studies have examined the impact of the anti-inflammatory diet and specific foods with potentially health-promoting effects, such as flaxseed [168]. 

There is currently a lack of studies on the use of the dietary approaches to stop hypertension (DASH) diet, which includes limiting salt intake, in patients with RA. In the Iranian population, a multicentre study showed that individuals with RA are less likely to adhere to the DASH diet compared with healthy individuals [169,170]. The authors of another interesting study in Iran examined the impact of the Mediterranean–DASH intervention for neurodegenerative delay (MIND) diet on the development and activity of RA [171]. This diet features a nutritional pattern that combines the Mediterranean and DASH diets; it stands out for its specific designated portions of berries and green leafy vegetables. Previous studies have shown that, in addition to reducing cardiovascular risk, adherence to the MIND diet is associated with improved cognitive function and a lower risk of developing Alzheimer’s disease [172]. Safaei et al. [171] demonstrated that adherence to the MIND diet may be associated with a lower risk of developing RA and reduced disease activity. As with other dietary interventions, further research is necessary to determine the exact relationship. However, the reduction in the DAS28 score observed in the Iranian study was significant.

Research has also focused on a high-residue diet. It has been shown that a high-fibre diet can positively affect the gut microbiome and reduce the risk of developing colorectal cancer [173]. In the USA, an analysis of patients from the NHANES database revealed that individuals with higher daily fibre intake are less likely to develop RA compared with those with a low fibre intake. Additionally, a high-fibre diet has a beneficial effect on reducing inflammation, which could potentially contribute to decreased RA activity [60]. There were similar conclusions in a German prospective study conducted from late 2018 to early 2019. After 28 days of fibre-rich supplementation, patients with RA who initially had low DAS28 scores experienced a reduction in markers of bone erosion and an increase in Treg count, while their quality of life improved [55]. Jiang et al. [67] emphasised that the role of dietary interventions in individuals with RA is limited when patients also have dysbiosis. They demonstrated in a mouse model of arthritis that a high-fibre diet combined with concurrent colonisation by *P. copri* led to exacerbation of RA. Table 2 summarises dietary interventions associated with an impact on RA progression. 

## 6. Conclusions

Overall, the current evidence suggests that gut dysbiosis is involved in the pathogenesis of RA. The composition of the intestinal microbiome and intestinal permeability has immunoregulatory properties, which affects the behaviour and differentiation of immune cells. The diversity and richness of the gut microbiome seems to be reduced in RA. Perhaps monitoring the gut microbiome could help in evaluating treatment response. Although we focused on bacteria, other components of the microbiome should also be investigated. Dietary factors and interventions have a marked impact in controlling the progression of RA. Lifestyle modifications involving dietary changes such as higher fibre intake and reduced consumption of red meat should be recommended for patients diagnosed with RA. However, further studies should examine the influence of specific dietary elements on the inflammatory parameters and mechanisms associated with RA. More studies are required to analyse the potential use of probiotics in patients with RA due to controversial results [174].

## Figures and Tables

**Table 1:** A summary of the various roles of different dietary elements on the processes associated with rheumatoid arthritis.

Dietary Element	Impact on Rheumatoid Arthritis	Mechanisms Associated with Rheumatoid Arthritis	References
High-fibre diet	Suppresses	Increasing in the number of Tregs. Increasing in the Th1/Th17 ratio. Decreasing IgA antibodies and anti-citrullinated vimentin p18 peptide antibody level. Reducing CTX-1 serum level. Protective role for the mucus in the intestine and intestinal barrier. SCFAs, metabolites of fibre, increase IL-10 production by B-cells and increase Treg cells.	[55,63,64,65,66]
Omega-3 fatty acids	Suppresses	Decrease in TNF-alpha, IL-1β, IL-6 and COX-2 metabolites. By replacing arachidonic acid in the cell membrane of the cells, they alter the gene transcription, cell signalling and metabolism of inflammatory mediators. Decreasing in adhesion molecules on the cell surfaces. Activating PPAR-gamma and inhibiting NF-kB. Interfering with raft formation in the inflammatory cell’s membrane via TLR 4 and myeloid differentiation primary response gene 88 (MyD88).	[68,69,71,72,73,74,81]
Vitamin D 3	Suppresses/no effect	Regulating tight junctions in the intestine and apoptosis of intestinal epithelial cells, contributing to maintaining gut barrier. Via downregulating TLR 2 and TLR 4 in monocytes, modulating the response to PAMPS and damage-associated molecular patterns (DAMPs). Suppressing Th17, Th1, follicular B helper T cells, and inhibiting secretion of IL-17. Promoting differentiation of Th2 cells and regulatory T cells. Suppressing DC maturation and antigen presentation cell surface antigens on monocyte-derived DCs. Decreasing expression of IFN-gamma, IL-21, IL-6, IL-2. Inhibiting autoantibody production by B cells. Promoting monocyte differentiation into macrophages, leading to reduced antigen presentation capacity by decreasing the expression of MHC II.	[87,88,92,93,94,95]
Vitamin E	Suppresses	Incorporating into the membrane of the immune cells, maintaining its integrity, and participating in transduction, cell division, and generation of inflammatory mediators. Scavenging of reactive oxygen species. Protecting polyunsaturated fatty acids in the cell membrane from oxidation, preventing their damage. Improving thymic T cell differentiation, lymphocyte proliferation, helper T cell activity, and phagocytic activity. Antioxidant effect on naďve T cells. Enhancing early events of T cell activation. Protective function on gut barrier integrity. Supporting favourable changes in gut microbial population, mitigating the depletion of butyrate-producing bacteria. Regulating function and maturation of DCs.	[102,103,114,115,116,117,118]
Selenium	Suppresses/no effect	The component of the active centre of antioxidant enzymes, among them glutathione peroxidase. Inhibiting NF-kB cascade. Regulating both innate and adaptive immune responses. Enhancing INF-gamma, supporting the differentiation and proliferation of T cells, and maintaining antibody levels. Being an immunostimulant, activating natural killer cells and cytotoxic lymphocyte-mediated tumour cytotoxicity. Scavenging ROS.	[119,124,127,128,129]
Red meat	Enhances/no effect	Glycation end products, which are formed during cooking the meat at high temperatures, increase inflammation. Free haems, a product of digestion, catalyse oxidative reactions damaging lipids, proteins, DNA and other nucleic acids. Sulphur in the meat is a source of energy for sulphate-reducing bacteria, which produce hydrogen sulphide contributing to the degradation of mucus, leaving the mucus layer exposed to bacterial penetration.	[63,133,134,135,136,137,138,139,140,141]
Saturated fats	Enhances	Promoting T cell activation and differentiation towards Th1 and Th17 cells. Activating STAT5-PI3K/Akt signalling pathway in T cells, which leads to the upregulation of signal lymphocyte-activating molecule family member 3, and proinflammatory cytokines such as TNF-alpha, IL-1β, IL-2, and IL-6. Inducing TNF-alpha expression in macrophages, activating TLR4 stimulating IL-8. Decreasing *Bifidobacteria*, *Eubacterium*, rectal *Clostridium coccoides* group, and *Bacteroides* in the intestines. Shifting to a dominance of Gram-negative bacteria in the intestine, rich in LPS, activating NF-kB. Triggering proinflammatory signalling pathways by interacting with the membrane receptors.	[144,145,146,147,148,150,151,153,154]

**Table 2:** Dietary interventions and their impact on RA.

Diet Type	Impact on RA (Enhance/Suppress)	Mechanism Associated with RA	References
High-fibre diet.	Suppresses, and in the case of dysbiosis, it worsens.	Reduces inflammation, improves quality of life; in the case of concurrent colonisation with *Prevotella copri*, it may lead to RA exacerbation.	[55,60,67]
Mediterranean diet.	Suppresses/no effect.	Positive effect on overall health (prevention of cancer and cardiovascular diseases), positive effect on mental health (prevention of depression), reduces or has no effect on RA activity.	[135,157,158,159]
Anti-inflammatory diet.	Suppresses/has no effect.	Reduces or has no effect on RA activity.	[167,168]
MIND diet.	Suppresses.	Prevention of cardiovascular diseases, improvement in cognitive function, reduces RA activity.	[171,172]
